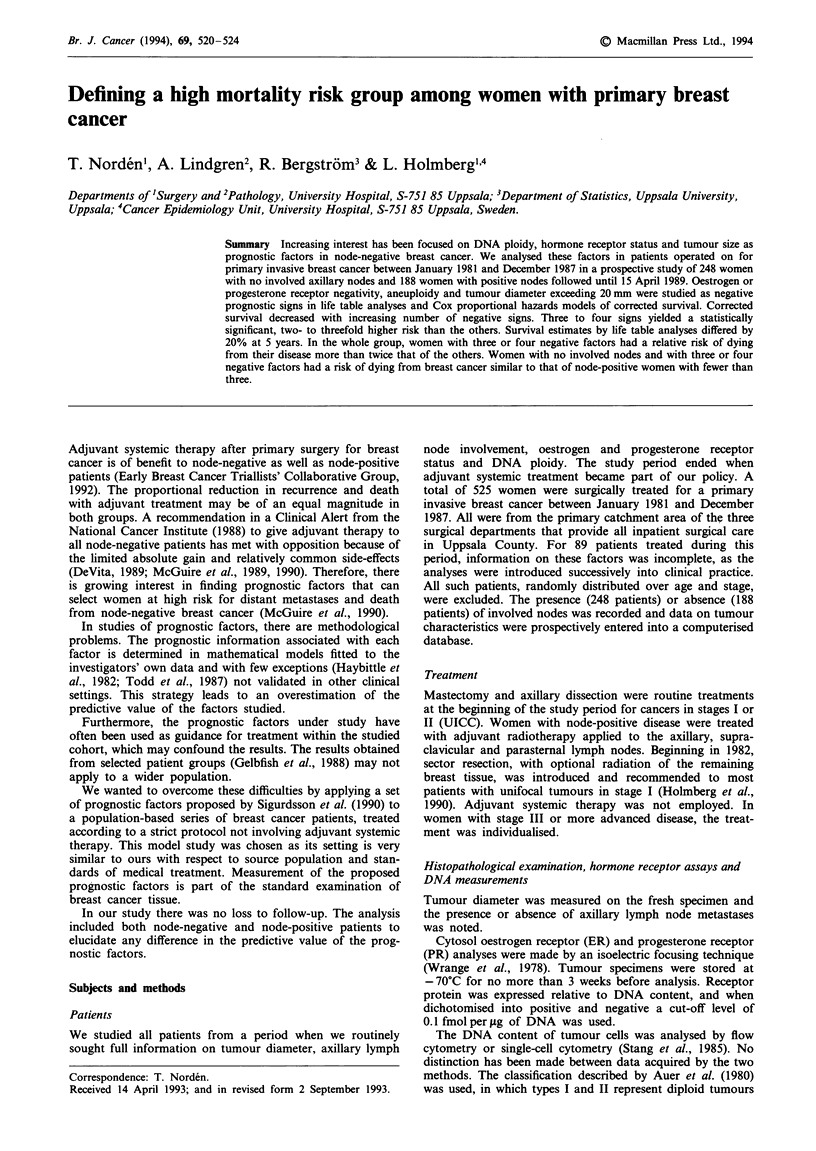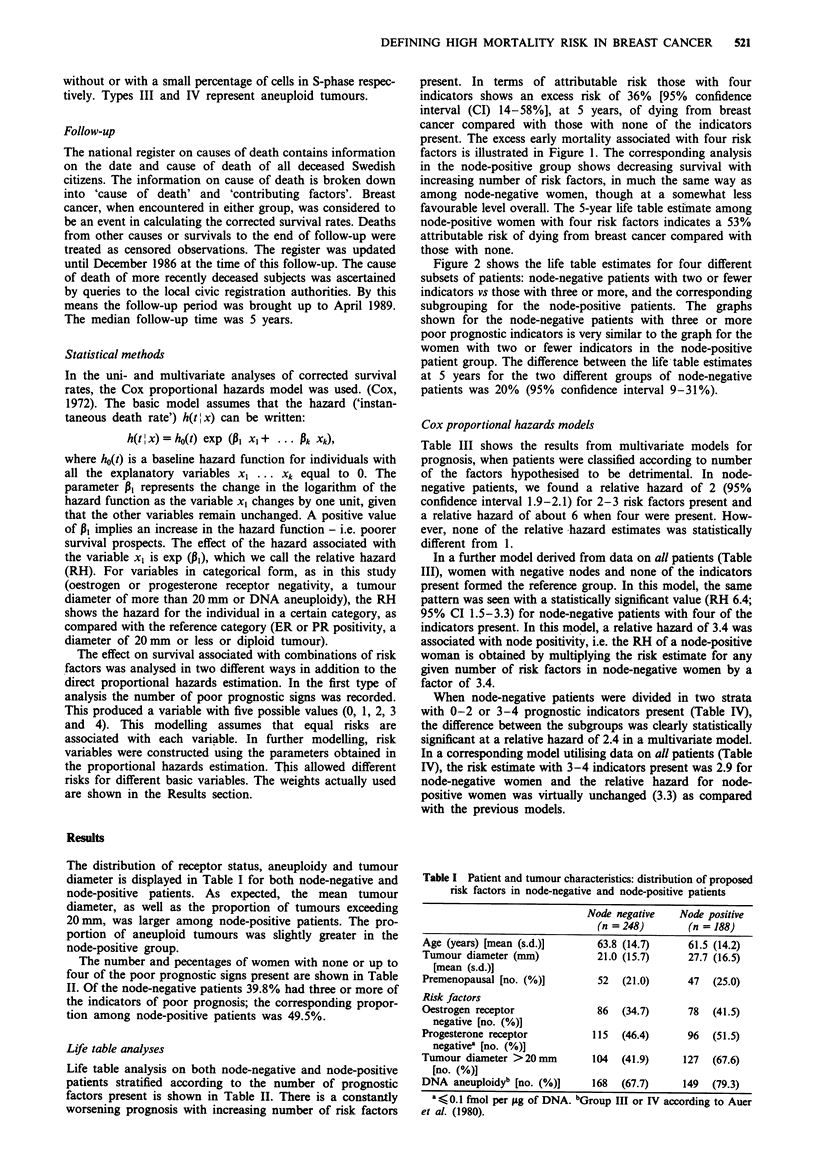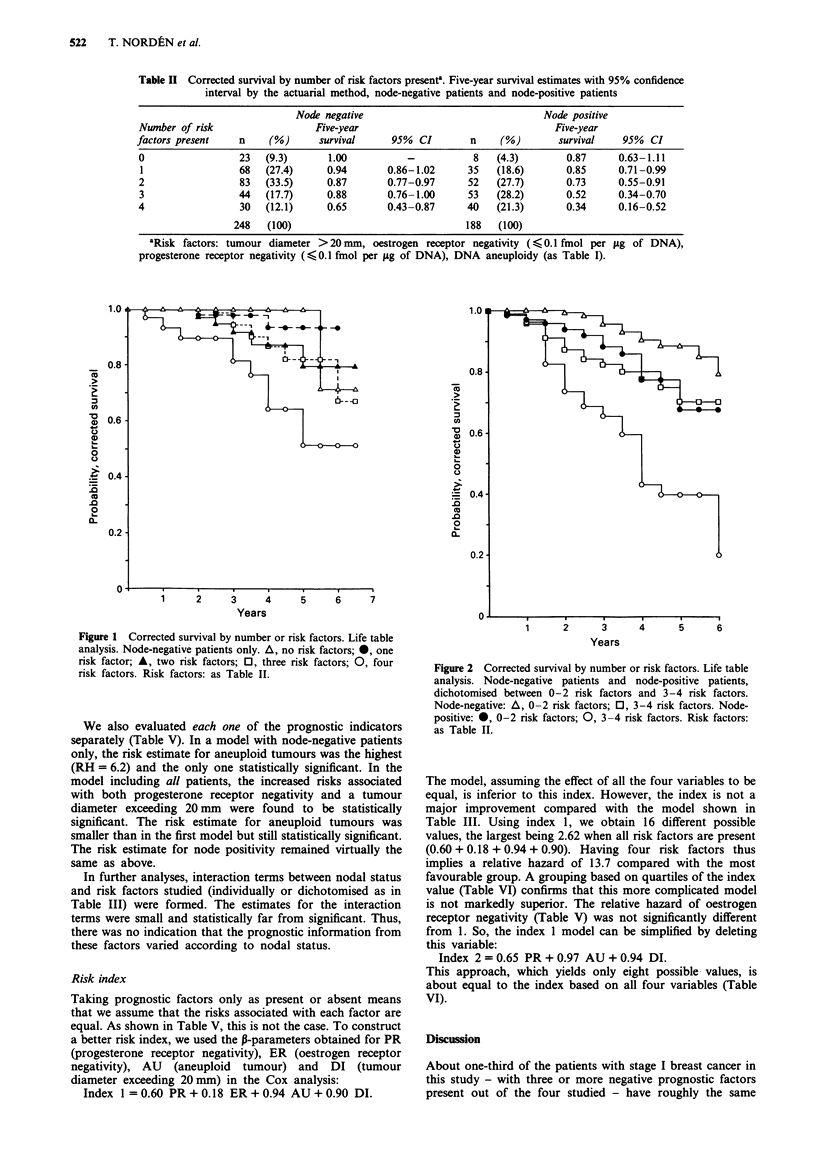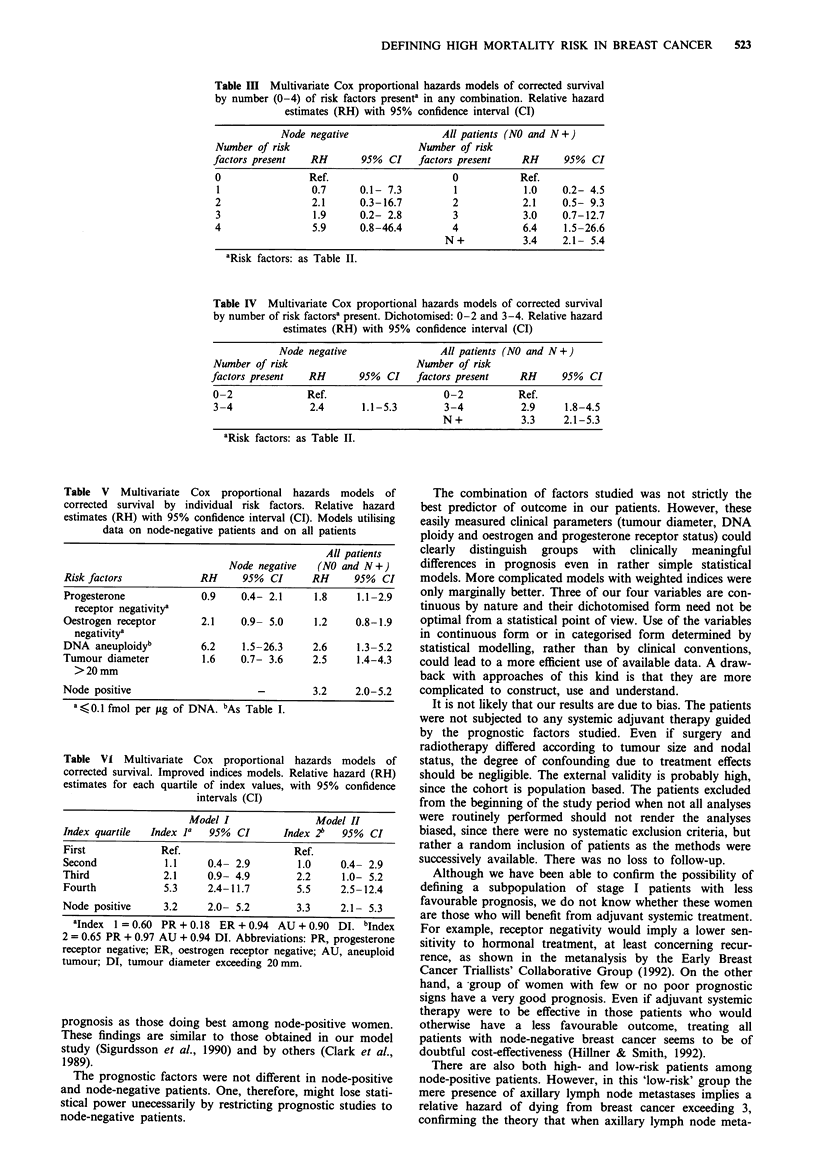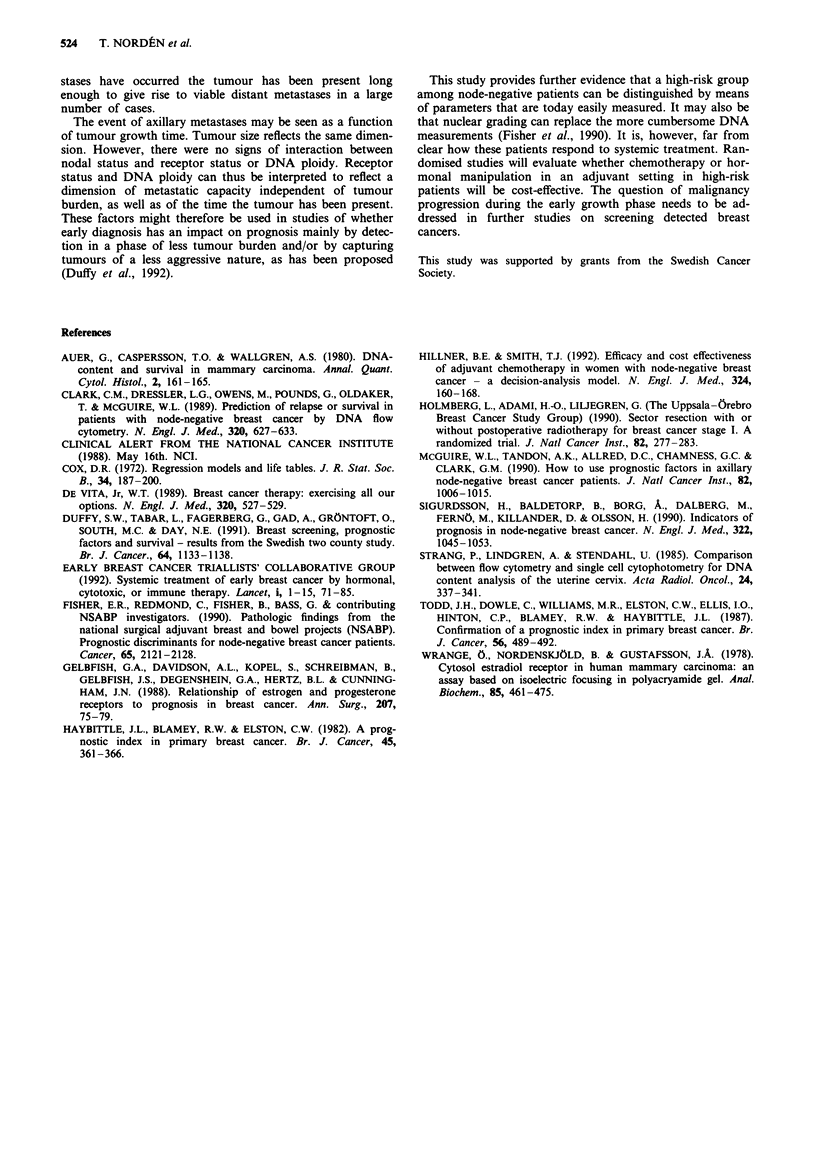# Defining a high mortality risk group among women with primary breast cancer.

**DOI:** 10.1038/bjc.1994.94

**Published:** 1994-03

**Authors:** T. Nordén, A. Lindgren, R. Bergström, L. Holmberg

**Affiliations:** Department of Surgery, University Hospital, Uppsala, Sweden.

## Abstract

Increasing interest has been focused on DNA ploidy, hormone receptor status and tumour size as prognostic factors in node-negative breast cancer. We analysed these factors in patients operated on for primary invasive breast cancer between January 1981 and December 1987 in a prospective study of 248 women with no involved axillary nodes and 188 women with positive nodes followed until 15 April 1989. Oestrogen or progesterone receptor negativity, aneuploidy and tumour diameter exceeding 20 mm were studied as negative prognostic signs in life table analyses and Cox proportional hazards models of corrected survival. Corrected survival decreased with increasing number of negative signs. Three to four signs yielded a statistically significant, two- to threefold higher risk than the others. Survival estimates by life table analyses differed by 20% at 5 years. In the whole group, women with three or four negative factors had a relative risk of dying from their disease more than twice that of the others. Women with no involved nodes and with three or four negative factors had a risk of dying from breast cancer similar to that of node-positive women with fewer than three.


Br. J. Cancer (1994), 69, 520–524 © Macmillan Press Ltd., 1994

### Defining a high mortality risk group among women with primary breast cancer

T. Nordén[1], A. Lindgren[2], R. Bergström[3] & L. Holmberg[1,4]

*Departments of [1]Surgery and [2]Pathology, University Hospital, S-751 85 Uppsala; [3]Department of Statistics, Uppsala University, Uppsala; [4]Cancer Epidemiology Unit, University Hospital, S-751 85 Uppsala, Sweden.*

**Summary** Increasing interest has been focused on DNA ploidy, hormone receptor status and tumour size as prognostic factors in node-negative breast cancer. We analysed these factors in patients operated on for primary invasive breast cancer between January 1981 and December 1987 in a prospective study of 248 women with no involved axillary nodes and 188 women with positive nodes followed until 15 April 1989. Oestrogen or progesterone receptor negativity, aneuploidy and tumour diameter exceeding 20 mm were studied as negative prognostic signs in life table analyses and Cox proportional hazards models of corrected survival. Corrected survival decreased with increasing number of negative signs. Three to four signs yielded a statistically significant, two- to threefold higher risk than the others. Survival estimates by life table analyses differed by 20% at 5 years. In the whole group, women with three or four negative factors had a relative risk of dying from their disease more than twice that of the others. Women with no involved nodes and with three or four negative factors had a risk of dying from breast cancer similar to that of node-positive women with fewer than three.

Adjuvant systemic therapy after primary surgery for breast cancer is of benefit to node-negative as well as node-positive patients (Early Breast Cancer Triallists' Collaborative Group, 1992). The proportional reduction in recurrence and death with adjuvant treatment may be of an equal magnitude in both groups. A recommendation in a Clinical Alert from the National Cancer Institute (1988) to give adjuvant therapy to all node-negative patients has met with opposition because of the limited absolute gain and relatively common side-effects (DeVita, 1989; McGuire *et al.*, 1989, 1990). Therefore, there is growing interest in finding prognostic factors that can select women at high risk for distant metastases and death from node-negative breast cancer (McGuire *et al.*, 1990).

In studies of prognostic factors, there are methodological problems. The prognostic information associated with each factor is determined in mathematical models fitted to the investigators' own data and with few exceptions (Haybittle *et al.*, 1982; Todd *et al.*, 1987) not validated in other clinical settings. This strategy leads to an overestimation of the predictive value of the factors studied.

Furthermore, the prognostic factors under study have often been used as guidance for treatment within the studied cohort, which may confound the results. The results obtained from selected patient groups (Gelbfish *et al.*, 1988) may not apply to a wider population.

We wanted to overcome these difficulties by applying a set of prognostic factors proposed by Sigurdsson *et al.* (1990) to a population-based series of breast cancer patients, treated according to a strict protocol not involving adjuvant systemic therapy. This model study was chosen as its setting is very similar to ours with respect to source population and standards of medical treatment. Measurement of the proposed prognostic factors is part of the standard examination of breast cancer tissue.

In our study there was no loss to follow-up. The analysis included both node-negative and node-positive patients to elucidate any difference in the predictive value of the prognostic factors.

### Subjects and methods

### *Patients*

We studied all patients from a period when we routinely sought full information on tumour diameter, axillary lymph node involvement, oestrogen and progesterone receptor status and DNA ploidy. The study period ended when adjuvant systemic treatment became part of our policy. A total of 525 women were surgically treated for a primary invasive breast cancer between January 1981 and December 1987. All were from the primary catchment area of the three surgical departments that provide all inpatient surgical care in Uppsala County. For 89 patients treated during this period, information on these factors was incomplete, as the analyses were introduced successively into clinical practice. All such patients, randomly distributed over age and stage, were excluded. The presence (248 patients) or absence (188 patients) of involved nodes was recorded and data on tumour characteristics were prospectively entered into a computerised database.

### *Treatment*

Mastectomy and axillary dissection were routine treatments at the beginning of the study period for cancers in stages I or II (UICC). Women with node-positive disease were treated with adjuvant radiotherapy applied to the axillary, supraclavicular and parasternal lymph nodes. Beginning in 1982, sector resection, with optional radiation of the remaining breast tissue, was introduced and recommended to most patients with unifocal tumours in stage I (Holmberg *et al.*, 1990). Adjuvant systemic therapy was not employed. In women with stage III or more advanced disease, the treatment was individualised.

### *Histopathological examination, hormone receptor assays and DNA measurements*

Tumour diameter was measured on the fresh specimen and the presence or absence of axillary lymph node metastases was noted.

Cytosol oestrogen receptor (ER) and progesterone receptor (PR) analyses were made by an isoelectric focusing technique (Wrange *et al.*, 1978). Tumour specimens were stored at −70°C for no more than 3 weeks before analysis. Receptor protein was expressed relative to DNA content, and when dichotomised into positive and negative a cut-off level of 0.1 fmol per μg of DNA was used.

The DNA content of tumour cells was analysed by flow cytometry or single-cell cytometry (Stang *et al.*, 1985). No distinction has been made between data acquired by the two methods. The classification described by Auer *et al.* (1980) was used, in which types I and II represent diploid tumours

Correspondence: T. Nordén.
Received 14 April 1993; and in revised form 2 September 1993.

without or with a small percentage of cells in S-phase respectively. Types III and IV represent aneuploid tumours.

*Follow-up*

The national register on causes of death contains information on the date and cause of death of all deceased Swedish citizens. The information on cause of death is broken down into 'cause of death' and 'contributing factors'. Breast cancer, when encountered in either group, was considered to be an event in calculating the corrected survival rates. Deaths from other causes or survivals to the end of follow-up were treated as censored observations. The register was updated until December 1986 at the time of this follow-up. The cause of death of more recently deceased subjects was ascertained by queries to the local civic registration authorities. By this means the follow-up period was brought up to April 1989. The median follow-up time was 5 years.

*Statistical methods*

In the uni- and multivariate analyses of corrected survival rates, the Cox proportional hazards model was used. (Cox, 1972). The basic model assumes that the hazard ('instantaneous death rate') $h(t|x)$ can be written:

$$h(t|x) = h_0(t) \exp(\beta_1 x_1 + \ldots \beta_k x_k),$$

where $h_0(t)$ is a baseline hazard function for individuals with all the explanatory variables $x_1 \ldots x_k$ equal to 0. The parameter $\beta_1$ represents the change in the logarithm of the hazard function as the variable $x_1$ changes by one unit, given that the other variables remain unchanged. A positive value of $\beta_1$ implies an increase in the hazard function – i.e. poorer survival prospects. The effect of the hazard associated with the variable $x_1$ is exp ($\beta_1$), which we call the relative hazard (RH). For variables in categorical form, as in this study (oestrogen or progesterone receptor negativity, a tumour diameter of more than 20 mm or DNA aneuploidy), the RH shows the hazard for the individual in a certain category, as compared with the reference category (ER or PR positivity, a diameter of 20 mm or less or diploid tumour).

The effect on survival associated with combinations of risk factors was analysed in two different ways in addition to the direct proportional hazards estimation. In the first type of analysis the number of poor prognostic signs was recorded. This produced a variable with five possible values (0, 1, 2, 3 and 4). This modelling assumes that equal risks are associated with each variable. In further modelling, risk variables were constructed using the parameters obtained in the proportional hazards estimation. This allowed different risks for different basic variables. The weights actually used are shown in the Results section.

### Results

The distribution of receptor status, aneuploidy and tumour diameter is displayed in Table I for both node-negative and node-positive patients. As expected, the mean tumour diameter, as well as the proportion of tumours exceeding 20 mm, was larger among node-positive patients. The proportion of aneuploid tumours was slightly greater in the node-positive group.

The number and pecentages of women with none or up to four of the poor prognostic signs present are shown in Table II. Of the node-negative patients 39.8% had three or more of the indicators of poor prognosis; the corresponding proportion among node-positive patients was 49.5%.

*Life table analyses*

Life table analysis on both node-negative and node-positive patients stratified according to the number of prognostic factors present is shown in Table II. There is a constantly worsening prognosis with increasing number of risk factors present. In terms of attributable risk those with four indicators shows an excess risk of 36% [95% confidence interval (CI) 14–58%], at 5 years, of dying from breast cancer compared with those with none of the indicators present. The excess early mortality associated with four risk factors is illustrated in Figure 1. The corresponding analysis in the node-positive group shows decreasing survival with increasing number of risk factors, in much the same way as among node-negative women, though at a somewhat less favourable level overall. The 5-year life table estimate among node-positive women with four risk factors indicates a 53% attributable risk of dying from breast cancer compared with those with none.

Figure 2 shows the life table estimates for four different subsets of patients: node-negative patients with two or fewer indicators *vs* those with three or more, and the corresponding subgrouping for the node-positive patients. The graphs shown for the node-negative patients with three or more poor prognostic indicators is very similar to the graph for the women with two or fewer indicators in the node-positive patient group. The difference between the life table estimates at 5 years for the two different groups of node-negative patients was 20% (95% confidence interval 9–31%).

*Cox proportional hazards models*

Table III shows the results from multivariate models for prognosis, when patients were classified according to number of the factors hypothesised to be detrimental. In node-negative patients, we found a relative hazard of 2 (95% confidence interval 1.9–2.1) for 2–3 risk factors present and a relative hazard of about 6 when four were present. However, none of the relative hazard estimates was statistically different from 1.

In a further model derived from data on *all* patients (Table III), women with negative nodes and none of the indicators present formed the reference group. In this model, the same pattern was seen with a statistically significant value (RH 6.4; 95% CI 1.5–3.3) for node-negative patients with four of the indicators present. In this model, a relative hazard of 3.4 was associated with node positivity, i.e. the RH of a node-positive woman is obtained by multiplying the risk estimate for any given number of risk factors in node-negative women by a factor of 3.4.

When node-negative patients were divided in two strata with 0–2 or 3–4 prognostic indicators present (Table IV), the difference between the subgroups was clearly statistically significant at a relative hazard of 2.4 in a multivariate model. In a corresponding model utilising data on *all* patients (Table IV), the risk estimate with 3–4 indicators present was 2.9 for node-negative women and the relative hazard for node-positive women was virtually unchanged (3.3) as compared with the previous models.

**Table I** Patient and tumour characteristics: distribution of proposed risk factors in node-negative and node-positive patients

| | *Node negative (n = 248)* | *Node positive (n = 188)* |
|---|---|---|
| Age (years) [mean (s.d.)] | 63.8 (14.7) | 61.5 (14.2) |
| Tumour diameter (mm) [mean (s.d.)] | 21.0 (15.7) | 27.7 (16.5) |
| Premenopausal [no. (%)] | 52 (21.0) | 47 (25.0) |
| *Risk factors* | | |
| Oestrogen receptor negative [no. (%)] | 86 (34.7) | 78 (41.5) |
| Progesterone receptor negative[a] [no. (%)] | 115 (46.4) | 96 (51.5) |
| Tumour diameter >20 mm [no. (%)] | 104 (41.9) | 127 (67.6) |
| DNA aneuploidy[b] [no. (%)] | 168 (67.7) | 149 (79.3) |

[a]⩽0.1 fmol per μg of DNA. [b]Group III or IV according to Auer *et al.* (1980).

**Table II** Corrected survival by number of risk factors present[a]. Five-year survival estimates with 95% confidence interval by the actuarial method, node-negative patients and node-positive patients

| Number of risk factors present | Node negative n | (%) | Five-year survival | 95% CI | Node positive n | (%) | Five-year survival | 95% CI |
|---|---|---|---|---|---|---|---|---|
| 0 | 23 | (9.3) | 1.00 | – | 8 | (4.3) | 0.87 | 0.63–1.11 |
| 1 | 68 | (27.4) | 0.94 | 0.86–1.02 | 35 | (18.6) | 0.85 | 0.71–0.99 |
| 2 | 83 | (33.5) | 0.87 | 0.77–0.97 | 52 | (27.7) | 0.73 | 0.55–0.91 |
| 3 | 44 | (17.7) | 0.88 | 0.76–1.00 | 53 | (28.2) | 0.52 | 0.34–0.70 |
| 4 | 30 | (12.1) | 0.65 | 0.43–0.87 | 40 | (21.3) | 0.34 | 0.16–0.52 |
| | 248 | (100) | | | 188 | (100) | | |

[a]Risk factors: tumour diameter >20 mm, oestrogen receptor negativity (⩽0.1 fmol per μg of DNA), progesterone receptor negativity (⩽0.1 fmol per μg of DNA), DNA aneuploidy (as Table I).

**Figure 1** Corrected survival by number or risk factors. Life table analysis. Node-negative patients only. △, no risk factors; ●, one risk factor; ▲, two risk factors; □, three risk factors; ○, four risk factors. Risk factors: as Table II.

**Figure 2** Corrected survival by number or risk factors. Life table analysis. Node-negative patients and node-positive patients, dichotomised between 0–2 risk factors and 3–4 risk factors. Node-negative: △, 0–2 risk factors; □, 3–4 risk factors. Node-positive: ●, 0–2 risk factors; ○, 3–4 risk factors. Risk factors: as Table II.

We also evaluated *each one* of the prognostic indicators separately (Table V). In a model with node-negative patients only, the risk estimate for aneuploid tumours was the highest (RH = 6.2) and the only one statistically significant. In the model including *all* patients, the increased risks associated with both progesterone receptor negativity and a tumour diameter exceeding 20 mm were found to be statistically significant. The risk estimate for aneuploid tumours was smaller than in the first model but still statistically significant. The risk estimate for node positivity remained virtually the same as above.

In further analyses, interaction terms between nodal status and risk factors studied (individually or dichotomised as in Table III) were formed. The estimates for the interaction terms were small and statistically far from significant. Thus, there was no indication that the prognostic information from these factors varied according to nodal status.

### *Risk index*

Taking prognostic factors only as present or absent means that we assume that the risks associated with each factor are equal. As shown in Table V, this is not the case. To construct a better risk index, we used the β-parameters obtained for PR (progesterone receptor negativity), ER (oestrogen receptor negativity), AU (aneuploid tumour) and DI (tumour diameter exceeding 20 mm) in the Cox analysis:

Index 1 = 0.60 PR + 0.18 ER + 0.94 AU + 0.90 DI.

The model, assuming the effect of all the four variables to be equal, is inferior to this index. However, the index is not a major improvement compared with the model shown in Table III. Using index 1, we obtain 16 different possible values, the largest being 2.62 when all risk factors are present (0.60 + 0.18 + 0.94 + 0.90). Having four risk factors thus implies a relative hazard of 13.7 compared with the most favourable group. A grouping based on quartiles of the index value (Table VI) confirms that this more complicated model is not markedly superior. The relative hazard of oestrogen receptor negativity (Table V) was not significantly different from 1. So, the index 1 model can be simplified by deleting this variable:

Index 2 = 0.65 PR + 0.97 AU + 0.94 DI.

This approach, which yields only eight possible values, is about equal to the index based on all four variables (Table VI).

### Discussion

About one-third of the patients with stage I breast cancer in this study – with three or more negative prognostic factors present out of the four studied – have roughly the same

**Table III** Multivariate Cox proportional hazards models of corrected survival by number (0–4) of risk factors present[a] in any combination. Relative hazard estimates (RH) with 95% confidence interval (CI)

| *Node negative* Number of risk factors present | RH | 95% CI | *All patients (N0 and N+)* Number of risk factors present | RH | 95% CI |
|---|---|---|---|---|---|
| 0 | Ref. | | 0 | Ref. | |
| 1 | 0.7 | 0.1– 7.3 | 1 | 1.0 | 0.2– 4.5 |
| 2 | 2.1 | 0.3–16.7 | 2 | 2.1 | 0.5– 9.3 |
| 3 | 1.9 | 0.2– 2.8 | 3 | 3.0 | 0.7–12.7 |
| 4 | 5.9 | 0.8–46.4 | 4 | 6.4 | 1.5–26.6 |
| | | | N+ | 3.4 | 2.1– 5.4 |

[a]Risk factors: as Table II.

**Table IV** Multivariate Cox proportional hazards models of corrected survival by number of risk factors[a] present. Dichotomised: 0–2 and 3–4. Relative hazard estimates (RH) with 95% confidence interval (CI)

| *Node negative* Number of risk factors present | RH | 95% CI | *All patients (N0 and N+)* Number of risk factors present | RH | 95% CI |
|---|---|---|---|---|---|
| 0–2 | Ref. | | 0–2 | Ref. | |
| 3–4 | 2.4 | 1.1–5.3 | 3–4 | 2.9 | 1.8–4.5 |
| | | | N+ | 3.3 | 2.1–5.3 |

[a]Risk factors: as Table II.

**Table V** Multivariate Cox proportional hazards models of corrected survival by individual risk factors. Relative hazard estimates (RH) with 95% confidence interval (CI). Models utilising data on node-negative patients and on all patients

| *Risk factors* | *Node negative* RH | *Node negative* 95% CI | *All patients (N0 and N+)* RH | *All patients (N0 and N+)* 95% CI |
|---|---|---|---|---|
| Progesterone receptor negativity[a] | 0.9 | 0.4– 2.1 | 1.8 | 1.1–2.9 |
| Oestrogen receptor negativity[a] | 2.1 | 0.9– 5.0 | 1.2 | 0.8–1.9 |
| DNA aneuploidy[b] | 6.2 | 1.5–26.3 | 2.6 | 1.3–5.2 |
| Tumour diameter >20 mm | 1.6 | 0.7– 3.6 | 2.5 | 1.4–4.3 |
| Node positive | | – | 3.2 | 2.0–5.2 |

[a]≤0.1 fmol per μg of DNA. [b]As Table I.

**Table VI** Multivariate Cox proportional hazards models of corrected survival. Improved indices models. Relative hazard (RH) estimates for each quartile of index values, with 95% confidence intervals (CI)

| *Index quartile* | *Model I* Index 1[a] | 95% CI | *Model II* Index 2[b] | 95% CI |
|---|---|---|---|---|
| First | Ref. | | Ref. | |
| Second | 1.1 | 0.4– 2.9 | 1.0 | 0.4– 2.9 |
| Third | 2.1 | 0.9– 4.9 | 2.2 | 1.0– 5.2 |
| Fourth | 5.3 | 2.4–11.7 | 5.5 | 2.5–12.4 |
| Node positive | 3.2 | 2.0– 5.2 | 3.3 | 2.1– 5.3 |

[a]Index 1 = 0.60 PR + 0.18 ER + 0.94 AU + 0.90 DI. [b]Index 2 = 0.65 PR + 0.97 AU + 0.94 DI. Abbreviations: PR, progesterone receptor negative; ER, oestrogen receptor negative; AU, aneuploid tumour; DI, tumour diameter exceeding 20 mm.

prognosis as those doing best among node-positive women. These findings are similar to those obtained in our model study (Sigurdsson *et al.*, 1990) and by others (Clark *et al.*, 1989).

The prognostic factors were not different in node-positive and node-negative patients. One, therefore, might lose statistical power unecessarily by restricting prognostic studies to node-negative patients.

The combination of factors studied was not strictly the best predictor of outcome in our patients. However, these easily measured clinical parameters (tumour diameter, DNA ploidy and oestrogen and progesterone receptor status) could clearly distinguish groups with clinically meaningful differences in prognosis even in rather simple statistical models. More complicated models with weighted indices were only marginally better. Three of our four variables are continuous by nature and their dichotomised form need not be optimal from a statistical point of view. Use of the variables in continuous form or in categorised form determined by statistical modelling, rather than by clinical conventions, could lead to a more efficient use of available data. A drawback with approaches of this kind is that they are more complicated to construct, use and understand.

It is not likely that our results are due to bias. The patients were not subjected to any systemic adjuvant therapy guided by the prognostic factors studied. Even if surgery and radiotherapy differed according to tumour size and nodal status, the degree of confounding due to treatment effects should be negligible. The external validity is probably high, since the cohort is population based. The patients excluded from the beginning of the study period when not all analyses were routinely performed should not render the analyses biased, since there were no systematic exclusion criteria, but rather a random inclusion of patients as the methods were successively available. There was no loss to follow-up.

Although we have been able to confirm the possibility of defining a subpopulation of stage I patients with less favourable prognosis, we do not know whether these women are those who will benefit from adjuvant systemic treatment. For example, receptor negativity would imply a lower sensitivity to hormonal treatment, at least concerning recurrence, as shown in the metanalysis by the Early Breast Cancer Triallists' Collaborative Group (1992). On the other hand, a group of women with few or no poor prognostic signs have a very good prognosis. Even if adjuvant systemic therapy were to be effective in those patients who would otherwise have a less favourable outcome, treating all patients with node-negative breast cancer seems to be of doubtful cost-effectiveness (Hillner & Smith, 1992).

There are also both high- and low-risk patients among node-positive patients. However, in this 'low-risk' group the mere presence of axillary lymph node metastases implies a relative hazard of dying from breast cancer exceeding 3, confirming the theory that when axillary lymph node metastases have occurred the tumour has been present long enough to give rise to viable distant metastases in a large number of cases.

The event of axillary metastases may be seen as a function of tumour growth time. Tumour size reflects the same dimension. However, there were no signs of interaction between nodal status and receptor status or DNA ploidy. Receptor status and DNA ploidy can thus be interpreted to reflect a dimension of metastatic capacity independent of tumour burden, as well as of the time the tumour has been present. These factors might therefore be used in studies of whether early diagnosis has an impact on prognosis mainly by detection in a phase of less tumour burden and/or by capturing tumours of a less aggressive nature, as has been proposed (Duffy *et al.*, 1992).

This study provides further evidence that a high-risk group among node-negative patients can be distinguished by means of parameters that are today easily measured. It may also be that nuclear grading can replace the more cumbersome DNA measurements (Fisher *et al.*, 1990). It is, however, far from clear how these patients respond to systemic treatment. Randomised studies will evaluate whether chemotherapy or hormonal manipulation in an adjuvant setting in high-risk patients will be cost-effective. The question of malignancy progression during the early growth phase needs to be addressed in further studies on screening detected breast cancers.

This study was supported by grants from the Swedish Cancer Society.

### References

AUER, G., CASPERSSON, T.O. & WALLGREN, A.S. (1980). DNA-content and survival in mammary carcinoma. *Annal. Quant. Cytol. Histol.*, **2**, 161–165.

CLARK, C.M., DRESSLER, L.G., OWENS, M., POUNDS, G., OLDAKER, T. & MCGUIRE, W.L. (1989). Prediction of relapse or survival in patients with node-negative breast cancer by DNA flow cytometry. *N. Engl. J. Med.*, **320**, 627–633.

CLINICAL ALERT FROM THE NATIONAL CANCER INSTITUTE (1988). May 16th. NCI.

COX, D.R. (1972). Regression models and life tables. *J. R. Stat. Soc. B.*, **34**, 187–200.

DE VITA, Jr, W.T. (1989). Breast cancer therapy: exercising all our options. *N. Engl. J. Med.*, **320**, 527–529.

DUFFY, S.W., TABAR, L., FAGERBERG, G., GAD, A., GRÖNTOFT, O., SOUTH, M.C. & DAY, N.E. (1991). Breast screening, prognostic factors and survival – results from the Swedish two county study. *Br. J. Cancer.*, **64**, 1133–1138.

EARLY BREAST CANCER TRIALLISTS' COLLABORATIVE GROUP (1992). Systemic treatment of early breast cancer by hormonal, cytotoxic, or immune therapy. *Lancet*, **i**, 1–15, 71–85.

FISHER, E.R., REDMOND, C., FISHER, B., BASS, G. & contributing NSABP investigators. (1990). Pathologic findings from the national surgical adjuvant breast and bowel projects (NSABP). Prognostic discriminants for node-negative breast cancer patients. *Cancer*, **65**, 2121–2128.

GELBFISH, G.A., DAVIDSON, A.L., KOPEL, S., SCHREIBMAN, B., GELBFISH, J.S., DEGENSHEIN, G.A., HERTZ, B.L. & CUNNINGHAM, J.N. (1988). Relationship of estrogen and progesterone receptors to prognosis in breast cancer. *Ann. Surg.*, **207**, 75–79.

HAYBITTLE, J.L., BLAMEY, R.W. & ELSTON, C.W. (1982). A prognostic index in primary breast cancer. *Br. J. Cancer*, **45**, 361–366.

HILLNER, B.E. & SMITH, T.J. (1992). Efficacy and cost effectiveness of adjuvant chemotherapy in women with node-negative breast cancer – a decision-analysis model. *N. Engl. J. Med.*, **324**, 160–168.

HOLMBERG, L., ADAMI, H.-O., LILJEGREN, G. (The Uppsala–Örebro Breast Cancer Study Group) (1990). Sector resection with or without postoperative radiotherapy for breast cancer stage I. A randomized trial. *J. Natl Cancer Inst.*, **82**, 277–283.

MCGUIRE, W.L., TANDON, A.K., ALLRED, D.C., CHAMNESS, G.C. & CLARK, G.M. (1990). How to use prognostic factors in axillary node-negative breast cancer patients. *J. Natl Cancer Inst.*, **82**, 1006–1015.

SIGURDSSON, H., BALDETORP, B., BORG, Å., DALBERG, M., FERNÖ, M., KILLANDER, D. & OLSSON, H. (1990). Indicators of prognosis in node-negative breast cancer. *N. Engl. J. Med.*, **322**, 1045–1053.

STRANG, P., LINDGREN, A. & STENDAHL, U. (1985). Comparison between flow cytometry and single cell cytophotometry for DNA content analysis of the uterine cervix. *Acta Radiol. Oncol.*, **24**, 337–341.

TODD, J.H., DOWLE, C., WILLIAMS, M.R., ELSTON, C.W., ELLIS, I.O., HINTON, C.P., BLAMEY, R.W. & HAYBITTLE, J.L. (1987). Confirmation of a prognostic index in primary breast cancer. *Br. J. Cancer*, **56**, 489–492.

WRANGE, Ö., NORDENSKJÖLD, B. & GUSTAFSSON, J.Å. (1978). Cytosol estradiol receptor in human mammary carcinoma: an assay based on isoelectric focusing in polyacryamide gel. *Anal. Biochem.*, **85**, 461–475.