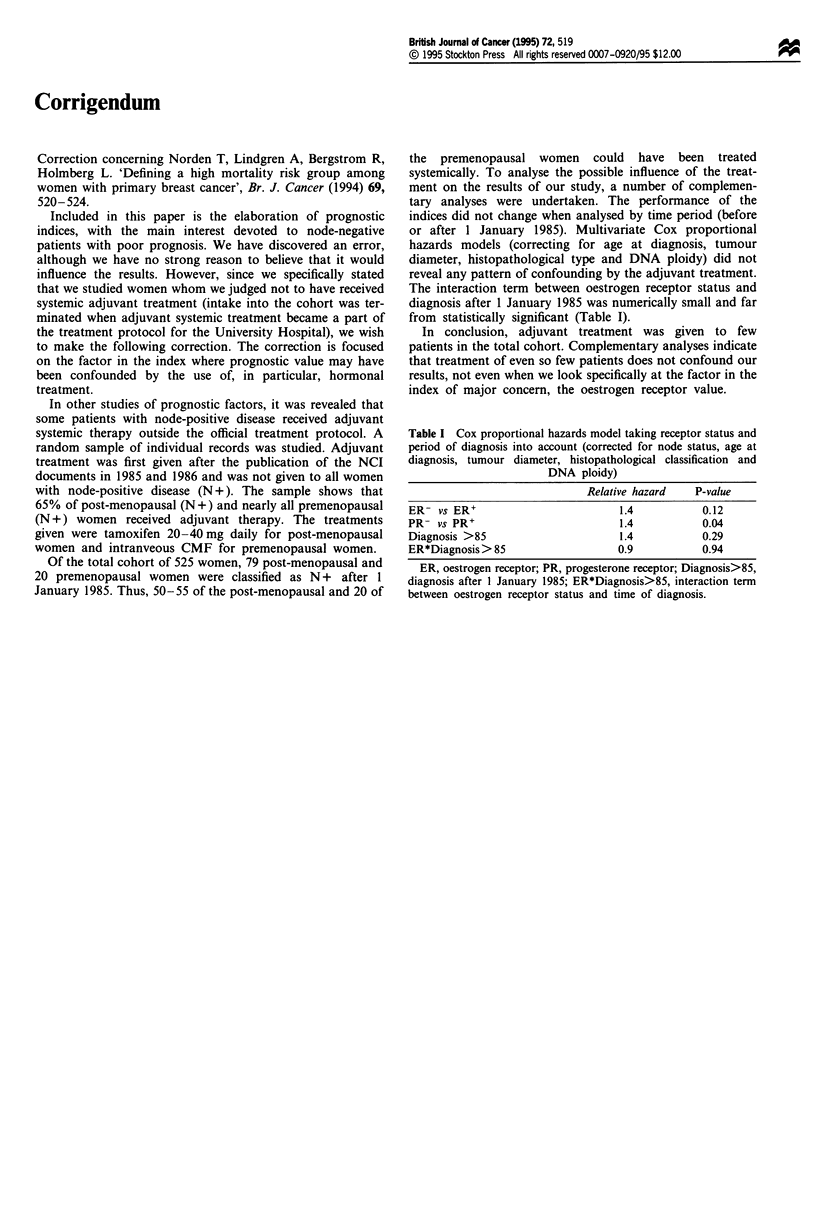# Corrigendum

**Published:** 1995-08

**Authors:** 


British Journal of Cancer (1995) 72, 519
© 1995 Stockton Press All rights reserved 0007-0920/95 $12.00

### Corrigendum

Correction concerning Norden T, Lindgren A, Bergstrom R, Holmberg L. 'Defining a high mortality risk group among women with primary breast cancer', *Br. J. Cancer* (1994) **69**, 520-524.

Included in this paper is the elaboration of prognostic indices, with the main interest devoted to node-negative patients with poor prognosis. We have discovered an error, although we have no strong reason to believe that it would influence the results. However, since we specifically stated that we studied women whom we judged not to have received systemic adjuvant treatment (intake into the cohort was terminated when adjuvant systemic treatment became a part of the treatment protocol for the University Hospital), we wish to make the following correction. The correction is focused on the factor in the index where prognostic value may have been confounded by the use of, in particular, hormonal treatment.

In other studies of prognostic factors, it was revealed that some patients with node-positive disease received adjuvant systemic therapy outside the official treatment protocol. A random sample of individual records was studied. Adjuvant treatment was first given after the publication of the NCI documents in 1985 and 1986 and was not given to all women with node-positive disease (N+). The sample shows that 65% of post-menopausal (N+) and nearly all premenopausal (N+) women received adjuvant therapy. The treatments given were tamoxifen 20-40 mg daily for post-menopausal women and intranveous CMF for premenopausal women.

Of the total cohort of 525 women, 79 post-menopausal and 20 premenopausal women were classified as N+ after 1 January 1985. Thus, 50-55 of the post-menopausal and 20 of the premenopausal women could have been treated systemically. To analyse the possible influence of the treatment on the results of our study, a number of complementary analyses were undertaken. The performance of the indices did not change when analysed by time period (before or after 1 January 1985). Multivariate Cox proportional hazards models (correcting for age at diagnosis, tumour diameter, histopathological type and DNA ploidy) did not reveal any pattern of confounding by the adjuvant treatment. The interaction term between oestrogen receptor status and diagnosis after 1 January 1985 was numerically small and far from statistically significant (Table I).

In conclusion, adjuvant treatment was given to few patients in the total cohort. Complementary analyses indicate that treatment of even so few patients does not confound our results, not even when we look specifically at the factor in the index of major concern, the oestrogen receptor value.

**Table I** Cox proportional hazards model taking receptor status and period of diagnosis into account (corrected for node status, age at diagnosis, tumour diameter, histopathological classification and DNA ploidy)

| | *Relative hazard* | P-*value* |
|---|---|---|
| $ER^-$ *vs* $ER^+$ | 1.4 | 0.12 |
| $PR^-$ *vs* $PR^+$ | 1.4 | 0.04 |
| Diagnosis >85 | 1.4 | 0.29 |
| ER*Diagnosis>85 | 0.9 | 0.94 |

ER, oestrogen receptor; PR, progesterone receptor; Diagnosis>85, diagnosis after 1 January 1985; ER*Diagnosis>85, interaction term between oestrogen receptor status and time of diagnosis.